# A new technique for ureteral resection to prevent urine dissemination and cancer seeding

**DOI:** 10.1002/iju5.12777

**Published:** 2024-08-26

**Authors:** Hayato Hoshina, Toru Sugihara, Masayuki Kurokawa, Kaori Endo, Ei‐ichiro Takaoka, Satoshi Ando, Haruki Kume, Tetsuya Fujimura

**Affiliations:** ^1^ Department of Urology Jichi Medical University Hospital Shimotsuke Japan; ^2^ Department of Urology, Graduate School of Medicine The University of Tokyo Bunkyo‐ku Japan

**Keywords:** laparoscopic radical nephroureterectomy (LNU), recurrence, robot‐assisted radical ureterectomy (RAU), transurethral resection of the bladder tumor (TURBT), upper tract urothelial cancer (UTUC)

## Abstract

**Introduction:**

We describe a case in which robot‐assisted radical ureterectomy was performed for residual ureteral recurrence in a postoperative patient with renal pelvis cancer.

**Case presentation:**

A 75‐year‐old woman underwent laparoscopic radical nephroureterectomy for left renal pelvic cancer at another hospital. During follow‐up, a papillary tumor was found in the bladder on cystoscopy, and a continuous tumor was found in the residual ureter on computed tomography. Robot‐assisted radical ureterectomy was performed in combination with transurethral resection of bladder tumors. To avoid urine spillage, bladder cuff excision was performed using a mechanical suture device while monitoring the interior of the bladder.

**Conclusion:**

We developed a new technique for remnant ureteral resection using cystoscopy and a vascular stapler to prevent urine dissemination and cancer seeding.

**Abbreviations & Acronyms**
CT: computed tomography
LNU: laparoscopic radical nephroureterectomy
RAU: robot‐assisted radical nephroureterectomy
TURBT: transurethral resection of the bladder tumor
UTUC: upper tract urothelial carcinoma

**Keynote message**

We believe that it is important not to open the urinary tract during bladder cuff excision to avoid urine and cancer cell dissemination. Here, we describe a case of robot‐assisted radical nephroureterectomy in a patient with recurrence in the residual ureter after laparoscopic radical nephroureterectomy. Bladder cuff excision was performed without opening the urinary tract using a mechanical suture device to prevent urine dissemination and cancer seeding.

## Introduction

Radical nephroureterectomy has long been the gold standard surgical treatment for UTUC.^1^, ^2^ Regarding the treatment of lower ureteric excision, three major approaches, whose goal is to cure cancer, have been reported in the literature.^3^, ^4^ The underlying factors of treatment include the degree of invasiveness and recurrence rate.^5^ Additionally, the standard UTUC surgical treatment involves specimen removal with en bloc bladder cuff excision.^6^ In addition to en bloc removal, it is important to avoid opening the urinary tract during bladder cuff excision. This prevents the seeding of tumor cells.

Here, we describe RAU combined with TURBT after LNU for UTUC that recurred in the residual ureter.

### Case presentation

**Fig. 1:**
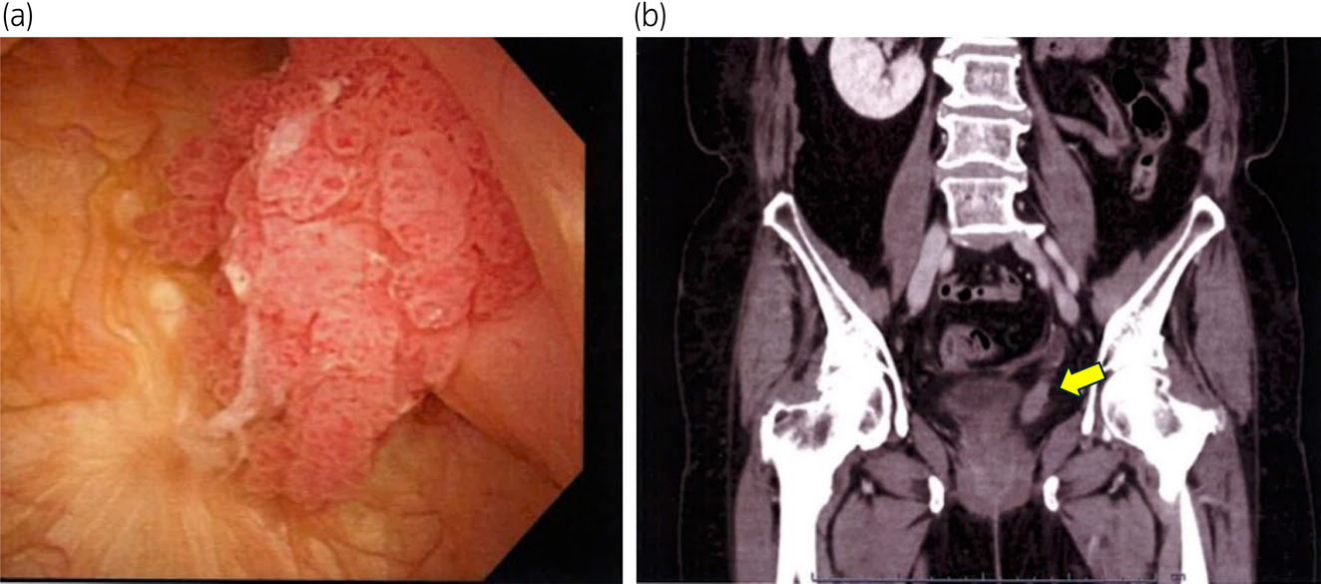
(a) A papillary tumor exiting the left ureteral orifice is seen on cystoscopy. (b) Tumor filling in the remaining ureter in the coronal view on CT (yellow arrow).

A 75‐year‐old woman underwent LNU for left renal pelvic cancer at another hospital. The pathology results showed urothelial carcinoma in situ, no vascular invasion, negative margins, and pT1N0M0 cancer. The patient was referred to our hospital for a follow‐up evaluation. Two years after follow‐up cystoscopy, recurrence of the papillary tumor was confirmed in the residual left ureteral orifice (Fig. 1a). Contrast‐enhanced CT revealed a tumor in the residual left ureter, extending into the bladder via the ureteral orifice (Fig. 1b).

Although the details of the previous surgery were unknown, CT showed that the remaining ureteral margin was visible up to the area around the common iliac artery crossing. Therefore, we decided to resect the papillary tumor visible through the left ureteral orifice by TURBT as much as possible and then perform robot‐assisted resection of the remaining ureter.

**Fig. 2:**
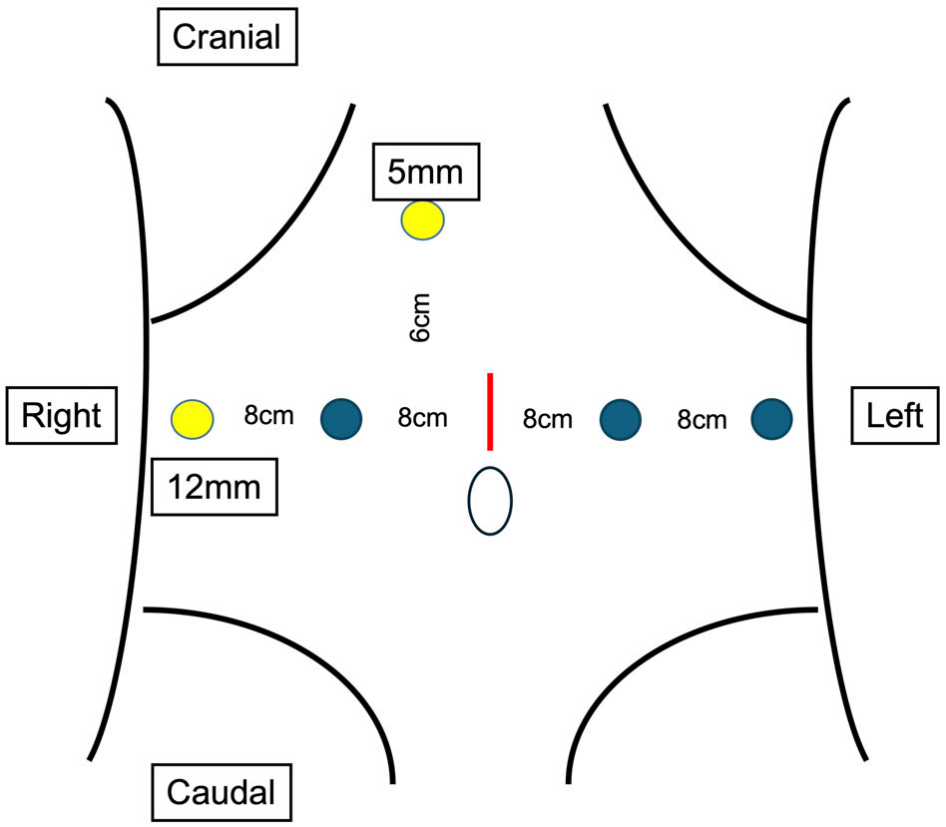
The camera port for the robot is placed near the umbilicus, 20 cm away from the pubis (red line). The left and right arms of the robot are placed 8 cm from the camera port in the lateral direction (blue circles). An extra arm is placed further left laterally 8 cm apart from the robotic left‐arm port. Five‐ and 12‐mm ports for an assistant surgeon are also placed 5–8 cm cephalically and right laterally, respectively, apart from the robotic right‐arm port (yellow circles).

**Fig. 3:**
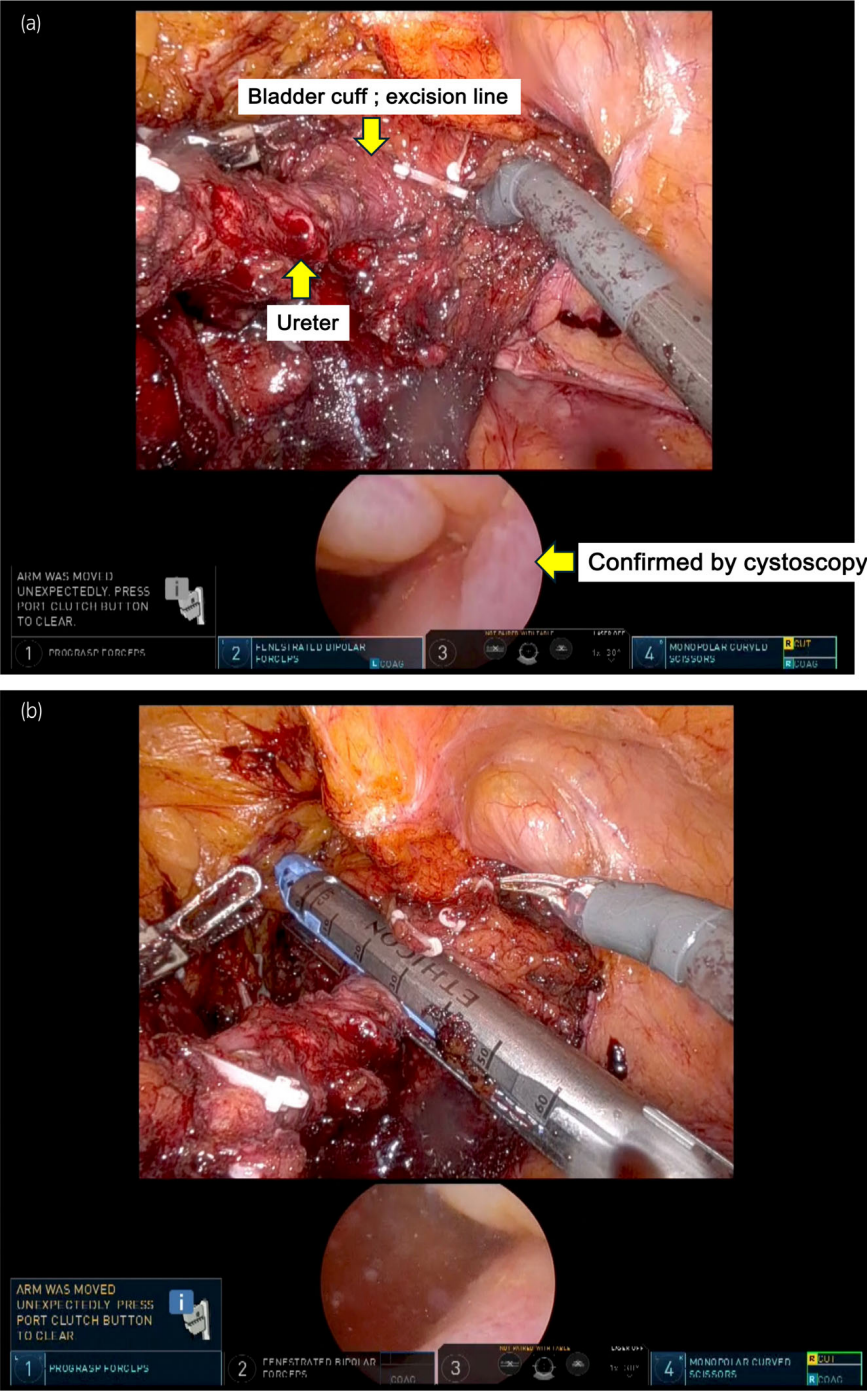
Confirmation that the ureter is fully stripped and extended (upper panel). Cystoscopy (lower panel) showing traction of the ureteral orifice (yellow arrow) (a) and bladder cuff excision using mechanical sutures (b).

The patient was placed in the lithotripsy position. As the left kidney had already been removed, there was no need to place the patient's upper body in the lateral position. First, the tumor located in the bladder was resected as much as possible by TURBT; thereafter, RAU was performed with the patient in the same position. The camera port for the robot was placed near the umbilicus, 20 cm away from the pubis. The robotic and assistant ports were arranged and set up, as shown in Figure 2. The surgery began with dissection of adhesions, and the site where the left ureter was cut off was explored. A clipped ureter was identified around the common iliac artery crossing, and the ureter was dissected toward bladder transition. The vesicoureteral junction area was widely dissected from the surrounding tissue and the bladder wall was retracted to form a tent‐shaped bladder cuff. Cystoscopy confirmed that the tent‐shaped bladder cuff involved the ureteral orifice. To remove the bladder cuff, including the mucosa and some muscle layers, from the bladder wall, a mechanical linear stapler (60 mm in length and 1.5 mm in height, Powered ECHELON FLEX®; Ethicon, Cincinnati, OH, USA) was used; this also prevented urinary spillage, which could cause intra‐abdominal seeding (Fig. 3a,b).

**Fig. 4:**
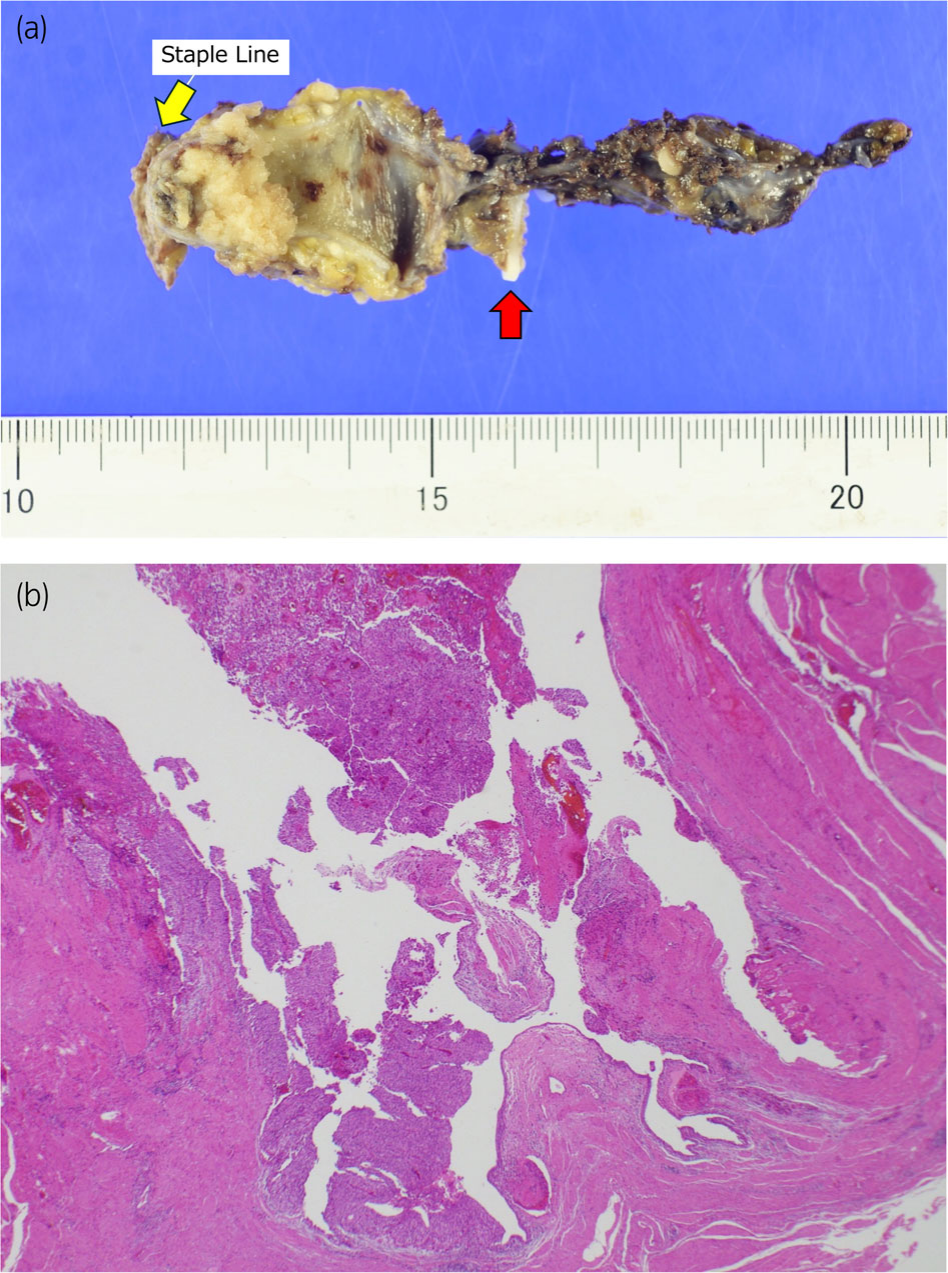
Specimen images. (a) Staple lines are indicated by yellow arrows. A clip of the previous nephroureterectomy is indicated by red arrows. A papillary tumor is recognized between them. (b) Pathological analysis of the specimen (using hematoxylin and eosin staining, ×20) shows the typical introversion characteristic of papillary tumors with no invasion into the stroma or muscularis.

The resection margins were negative, and the pathological diagnosis was urothelial carcinoma (pTa) (Fig. 4a,b). The patient was followed up, and postoperative cystoscopy showed no evidence of recurrence.

## Discussion

There has been much discussion regarding the method of bladder cuff resection for a long time. Three approaches have been reported: transvesical, extravesical, and transurethral.^3^, ^4^, ^5^ The oncological outcomes of each technique, as well as their advantages and disadvantages, have also been discussed. In the present case, we found that half of the left ureter had been left unremoved, which implies that the left ureter would have been cut‐off halfway through without verifying around the bladder because of renal pelvic cancer in the previous nephroureterectomy. Previous reports have shown that the recurrence of residual ureter is very high, and failure to excise the bladder cuff is associated with recurrence rates of 33–75% in the ureteric remnant.^6^ It is important to form a tent‐shaped bladder wall before cutting the bladder cuff and performing en bloc removal of the specimen involving the total ureter.

We also considered that opening the urinary tract would risk cancer seeding.^7^ Therefore, we performed bladder cuff excision using mechanical sutures to ensure that the bladder was sufficiently suspended in a tent‐like shape. This prevents urine leakage into the body. To avoid the concern that the ureteral orifice could remain unremoved after bladder cuff excision because of incomplete expansion, we ensured that the bladder orifice was completely included in the resected specimens using intraoperative cystoscopy.

One of the difficulties in using staplers is the assessment of a cut‐off margin. To better assure a negative margin, we performed broad TURBT from inside the bladder in this case. Additionally, it has been reported that postoperative pain and blood loss are less and that recovery is faster with the laparoscopic approach than with the open approach.^8^

Once a surgical operation is performed, the postoperative region is subjected to adhesions and requires a highly skilled technique while avoiding unexpected complications. Not only ureteral transection recurrence but also recurrence in the surrounding area due to scattered urine is important for prevention. Moreover, it is necessary to follow up with patients in the mid‐ to long‐term for the prevalence of cancer and the occurrence of infections and stone formation when the stapler slips out into the bladder. There are several reports on the use of staplers and stone formation.^9^, ^10^ A stapler is also used in the ileal conduit for urinary tract conversion surgery; however, stone formation has never been observed. The previous literature suggests that no complications, such as stones or infection, had occurred with the use of titanium staplers.^11^

## Conclusions

We described a case of RAU using a cystoscope in a patient with recurrent cancer in the residual ureter after LNU. In radical nephroureterectomy, the rate of cancer recurrence in the residual ureter is high; therefore, bladder cuff excision is an important procedure. Additionally, the risk of cancer recurrence in the surrounding area is reduced by the lack of urinary tract release during cuff excision. This new technique for remnant ureteral resection using cystoscopy and a vascular stapler prevents urine dissemination and cancer seeding.

## Author contributions

Hayato Hoshina: Conceptualization; data curation; formal analysis; investigation; methodology; project administration; writing – original draft. Toru Sugihara: Methodology; writing – review and editing. Masayuki Kurokawa: Writing – review and editing. Kaori Endo: Writing – review and editing. Ei‐ichiro Takaoka: Writing – review and editing. Satoshi Ando: Writing – review and editing. Haruki Kume: Writing – review and editing. Tetsuya Fujimura: Project administration; supervision; writing – review and editing.

## Conflict of interest

The authors declare no conflicts of interest.

## Approval of the research protocol by an Institutional Reviewer Board

The protocol for this research project was approved by the Institutional Review Board of Jichi Medical University Hospital (approval number: A22‐023).

## Informed consent

Written informed consent was obtained from the patient for publication of this case and accompanying images.

## Registry and the Registration No. of the study/trial

Not applicable.

## Data Availability

All data generated or analyzed during this study are included in this article. Further inquiries can be directed to the corresponding author.
